# When counting cattle is not enough: multiple perspectives in agricultural and veterinary research

**DOI:** 10.1186/1751-0147-53-S1-S6

**Published:** 2011-06-20

**Authors:** Bjørn Gunnar Hansen, Vidar Schei, Arent Greve

**Affiliations:** 1 Department of Strategy and Management, Norwegian School of Economics and Business Administration, Norway

## Abstract

A traditional approach in agricultural and veterinary research is focussing on the biological perspective where large cattle-databases are used to analyse the dairy herd. This approach has yielded valuable insights. However, recent research indicates that this knowledge-base can be further increased by examining agricultural and veterinary challenges from other perspectives. In this paper we suggest three perspectives that may supplement the biological perspective in agricultural and veterinary research; the economic-, the managerial-, and the social perspective. We review recent studies applying or combining these perspectives and discuss how multiple perspectives may improve our understanding and ability to handle cattle-health challenges.

## Introduction

Agricultural and veterinary-related problems rarely take place in isolation. On the contrary, when farmers call for help on herd health management issues, the challenges may be highly embedded in the economic, managerial, and social contexts of the farm. For example, knowing the economic status of the farm, the way farmers think, and the working climate at the farm may be valuable when we address issues as cattle-health challenges. Many agricultural and veterinary-related problems may therefore be better understood and handled when we have rich information about the context in which these problems occur.

However, despite early research showing the potential of adopting alternative perspectives [1], the traditional approach in agricultural and veterinary research has been a focus on *biological perspectives*. A widely used approach in veterinary and agricultural research is to address biological questions by analysing large cattle databases [2,3]. The analysis of data from the cattle database can be helpful in detecting what has happened in the dairy herd. A typical example is a cross sectional study of number of mastitis treatments per cow. This may reveal that number of treatments per cow decreases with increasing herd size, and the skilled researcher is able to come up with some possible explanations. However, the study also leaves the researcher with a lot of new questions, e.g.: “What are the economic implications of this finding?” “To what extent do managerial differences influence the number of treatments?” and “Can the findings be explained by the social interactions among the people at the farm?”

Thus, although studies of cattle databases are helpful in establishing how (mainly) biological variables are related, expanding the analysis to other perspectives and methodological approaches may help us better understand why variables interact. The purpose of this paper is to present three perspectives that may supplement the biological perspective in agricultural and veterinary research; the economic-, the managerial-, and the social perspective. We review recent studies applying these perspectives (see Table 1 for a brief overview), provide some illustrative examples of how the perspectives can be combined with the biological perspective, and discuss how different perspectives may improve our understanding of cattle-health challenges.

**Table 1:** Studies of Herd Related Issues that have Applied Biological, Economic and Managerial Perspectives

Study	Main perspective	Subject	Method
Hansen, Hegrenes, Larsen, Sehested, & Stokstad, 2005	Biological Economic	Financial impact of technical herd performance indicators	Mathematical and statistical modelling
Kristensen, Østergaard, Krogh, & Enevoldsen, 2007	Biological Economic	Financial impact of technical herd performance indicators	Mathematical modelling
Kristensen & Enevoldsen, 2008	Managerial	Farmers’ goals, beliefs attitudes and behaviour	Interviews and survey
Kristensen, Nielsen, Jensen, Vaarst, & Enevoldsen, 2008	Biological Managerial	Risk for metritis. Veterinarians perceptions and decision making	Interviews combined with quantitative analysis of herd data
Jansen, van den Borne, Renes, van Schaik, Lam, & Leeuwis, 2009	Biological Managerial	Farmers’ attitudes and behaviour	Survey
Lastein, Vaarst, & Enevoldsen, 2009	Managerial	Veterinarians’ perceptions and decision making	Field observations and interviews
Vaarst & Tind Sørensen, 2009	Biological Managerial	Dairy farmers’ perceptions and attitudes related to calf management	Semi-structured interviews
Van Asseldonk, Renes, Lam, & Hogeveen, 2010	Managerial	Farmers’ perceptions, intentions, actions and decision makings	Interviews

## The economic perspective

The economic perspective focuses on how data from accounting and performance measures may help farmers and veterinarians to better understand and act upon their biological data. Biological datasets lack information on prices, revenues and costs, and can only be used for calculating partial productivity indices such as milk yield per cow and herd fertility. To sustain the farm in the long run, farmers need at least to consider their costs and revenues, regardless of what is their primary reason for being a farmer. Supplementing cattle databases with economic data indicates the profitability of the biological production. In addition to biological productivity measures such as number of veterinary treatments per cow, economic data add the possibility of calculating economic performance measures such as gross margin (revenues deducted variable costs) per cow or net farm income (all revenues minus all expenses).

Economic data can be collected from different sources. An obvious source of economic data is the farm accountancy. For example, in Norway, TINE Efficiency Analysis (TEA) merges data from the cattle database and the farm accountancy. The farm bookkeeper sends selected accountancy data to a database, and the consultant in TINE SA adds some additional information on feeding of young stock, use of acreage, etc. The consultant calculates several productivity and profitability measures and delivers reports to the farmer once a year. Results are stored in the central cattle database along with data from comparable farms. TINE SA offers a follow-up either on the farm or in a workshop together with other farmers. Approximately 1800 Norwegian dairy farmers take part in this service every year. Thus, economic data are available, but are these data used to supplement biological data from cattle databases?

There are a few studies that combine the biological and the economic perspectives (see Table 1). For example [4] tried to establish links between technical performance indicators in dairy herds (e.g. shape of lactation curve, heifer management, reproduction efficiency) and their effects on gross margin per cow. They used a mathematical model which allowed estimation of the financial value of specific changes within the key performance indicators. The study indicates that improving the shape of the herd level lactation curve leads to an increase in gross margin per cow per year. The model can be manipulated by varying the technical key performance indicators to forecast the financial consequences of different “what-if” herd management options.

In [5] (Table 1), we used data from the TINE Efficiency Analysis discussed above and adopted an efficiency approach. While traditional economic performance measures compare only two aspects at a time (for example gross margin per litre milk), the efficiency approach can be used to evaluate the farms in a comprehensive way because all inputs and outputs can be considered at the same time [6]. The term efficiency is commonly defined as the minimum resource level that is theoretically required to run the desired operations in a given system compared to how much resources that are actually used [7]. A frequently used method for calculating efficiency is data envelopment analysis (DEA) [6]. DEA is a method for analysing relative efficiency when there are several outputs and inputs. We calculated an efficiency index for each farm. By using DEA we ranked the farms according to their relative economic efficiency, with one as maximum efficiency. Since the ranking is relative, the measure depends on the efficiency of the farms in the actual sample. The farms were ranked by DEA-analysis according to how high gross margin they produced given three different inputs; acreage of land, cowshed capacity and milk quota.

The ranking coefficient was used as the dependent variable in a regression model with approximately 80 relevant biological and economic explanatory variables. After finalising the analysis, the study resulted in a list of approximately 13 variables that explain why some farms are more efficient than others. A selection of the variables (e.g., total roughage costs, milk delivered of quota, heifers age) are provided as an example for one farm in Table 2. The table shows the results for one farm for the two last years together with a reference group. The reference group is useful for benchmarking, and represents an average of farms of similar size localised in an area with comparable production conditions. Heifers age is one of the variables that is important in predicting economic efficiency. Heifers at the actual farm calved when they were 27.8 months old in 2008, an increase from 25.0 months in 2007. Compared to the reference group the current farm is worse off, and should consider taking measures in order to regain the position it had in 2007. Similarly, insemination costs have increased from 0.10 in 2007 to 0.14 in 2008, which is well above the costs of the reference group.

**Table 2:** Selected Variables Predicting Farm Economic Efficiency

Key Performance Indicator	Unit	Farm 2007	Farm 2008	Reference group 2008
Total roughage costs	NOK/litre milk	1.69	1.59	1.96
Milk income – feed costs	NOK/litre milk	2.43	2.69	2.65
Fertility status	Number	21	26	n.a.
Heifers age at first calving	Months	25.0	27.8	26.4
Insemination costs	NOK/litre milk	0.10	0.14	0.11
Veterinary/ medicine costs	NOK/litre milk	0.08	0.06	0.11

The fact that the study finds a combination of both biological and economic key performance indicators points to the importance of including both types of data. An alternative model consisting of purely biological data reached only half the explanatory power compared to the model referred above. However, despite having access to an enlarged dataset, many questions are still left unanswered. For example, the farmer might ask: “How do I reduce my veterinary costs per litre milk?” Confronted with such questions the analysis of cattle and economic databases offers very little, if any, information on how farmers with low veterinary costs manage their herd. In order to obtain a better understanding there is a need to get behind the figures. Hence, we need other perspectives.

## The managerial perspective

Cattle and economic databases traditionally contain sparse information about managing the farm. This has to do with several aspects. First, the data are often collected for other purposes than guidance, such as breeding programs, farm accountancy and taxation. Second, the people who collect these data, have their specific professional focus and pay attention to particular aspects when visiting a farm—a construction engineer typically pays attention to the cowshed while a veterinarian is more concerned with the cows and how healthy they are. Thus, the role of the dairy farmer is often neglected or undervalued in farm research [8]. To obtain a better understanding of the causes of different events at the farm, one step forward is to merge biological data and economic data with data regarding the farmer as a manager. The managerial perspective focuses on the farmer’s perceptions and behaviour. More specifically, this perspective is about how the farmer makes and implements decisions.

The managerial perspective has been applied in several recent studies (see Table 1 for a summary). Some studies have the managerial aspect as their primary focus. For example, [9] investigated the behavioural and motivational sides of farmers’ expectations related to dairy herd health management. The farmers’ views could be classified in four categories: Teamwork, animal welfare, knowledge dissemination, and production. Interestingly, veterinarians believed that farmers’ primary focus is on production and profit, while in fact farmers valued teamwork and animal welfare more. Also looking at farmers perceptions, [10] examined the awareness, intentions and stated actions of farmers in reducing somatic cell count. They hypothesised that economic information can make farmers more aware of the importance of somatic cell count. The majority of the dairy farmers perceived projected economic losses due to elevated somatic cell count as not very relevant to them. The farmers justified their actions with regard to somatic cell count control in terms of their intention to manage the problem and their beliefs in whether their efforts would be successful. They rationalised their actions in a specific context connecting their intentions to their beliefs to produce a desired outcome. Other studies have examined the perceptions of veterinarians’ [11]. [11] showed how veterinarians’ motivations and decisions about medical treatment affected the quality of data recorded from the herd. Veterinarians’ focus on specific problems influenced their motivation to collect data and this may have introduced variation and bias into the data.

Other studies have—more explicitly—combined the managerial and the biological perspective. [12] focused on farmers’ time planning and structure of everyday activities as keys to understanding and solving problems related to calf disease and mortality. Whether the manager had a basic belief that calf mortality was a permanent crisis that had to be expected to be present on a dairy farm affected calf mortality in the herd. Previous experience in solving disease and mortality problems in calves had a strong positive influence on this belief. Furthermore, [13] reported that farmers’ normative frame of reference about mastitis explain variation in herd level somatic cell count and their perceptions of the control of mastitis. Thus, farmers’ perceptions about mastitis control explained the variation in clinical mastitis. The study concludes that farmers’ self-reported behaviour explain and predict differences in mastitis incidence between farms. Consequently, future research should take into account not only farmers’ behaviour but also farmers’ attitudes and how these may direct their perceptions and actions.

Some studies also point to the importance of combining various methodological approaches (e.g., [14]). [14] proposed that research in health management using solely a quantitative approach may present major challenges to the interpretation of the results, because the humans involved may respond to their observations based on previous experiences and their own beliefs. They concluded that drawing conclusions from quantitative studies that show associations between specific herd health management routines and disease outcome may benefit greatly by adding a qualitative perspective to the quantitative approach to reduce response bias.

Results from an on-going project seem to support the importance of using different methods as advocated by [14]. Preliminary analysis from this project shows a positive effect of the farmer being proactive in their problem solving and herd fertility. Proactive problem solving implies a preference to look for problems and to keep control of the situation, instead of giving up control and wait for problems to emerge on their own [15]. However, what strikes us as important from the latter project is not only the value of combining the managerial (proactive problem solving) and biological (herd fertility) perspectives, but the power of the methodological approach. The use of a semi-structured interview could capture the interviewees’ thought processes, the frame of reference, and the feelings about an incident or set of incidents which have meaning for the respondent (cf. [16]). According to [17] it is the way people constitute their reality and the way they view the world which shapes their future actions. To understand people’s actions it is important to map the way they think. Listening to the farmers talking about their problems, some of them spent a lot of the interview explaining how they worked to prevent problems from arising. Through detailed analyses (attributional coding) of the interview transcripts farmers that used proactive problem solving techniques could be identified. Although this method is time-consuming, a phenomenon such as farmers’ problem solving abilities cannot be adequately captured in a questionnaire. During the interview the farmers talked about their farming and problem solving with ease and appreciated being allowed to do so in their own words. In turn, these words can be very valuable data in agricultural and veterinary research.

## The social perspective

As is evident from the managerial perspective discussed above, the farmer clearly plays a crucial role managing the farm. Often however, the farmer is not running the farm alone. Thus, understanding the social context in which the farmer operates may be of vital importance to understand how agricultural and veterinary challenges are handled. The social perspective is about the interactions and relationships between those being involved in the daily farming activities and decision making. Thus, the social perspective includes phenomena such as communication, information exchange, conflict and conflict management, and team dynamics.

The social perspective is rarely used in the farming literature up until now. None of the studies listed in Table 1 used this approach. The absence of the social perspective in agricultural and veterinary research may be surprising in light of the findings in [9]. In their study of farmers’ expectations related to dairy herd health management, teamwork was one of the aspects that farmers valued the most. On the other hand, maybe the lack of research on teamwork should not be surprising as the veterinarians in the same study believed production and profit to be the farmers’ primary focus. Given the scarcity of agricultural and veterinary research adopting a social perspective, we next illustrate—by briefly reporting on a project in progress—how the perspective may possibly add value to the other perspectives.

In order to explore the social perspective we studied several collaborative farming projects. In Norway it has become quite popular for farmers to enter collaborative farming joining their quotas, herds and farming land. We interviewed two farmers in each joint operation, one at a time. Altogether we interviewed sixteen farmers in eight operations in two different regions. The interviews lasted from one to two hours and we emphasised factors that promote or hamper collaboration in the farmer team. Each joint operation has typically three or four active farmers. We are now analysing the interviews and the production data and plan to include more economic data. As an early analysis, we classified the quality of the teamwork as good, medium or poor. Similarly, experts in the different fields assessed how well the operations performed with respect to milk quality, animal health and herd fertility.

Preliminary analyses indicate a relationship between milk quota filling and quality of teamwork. Teams with good teamwork manage in general to deliver a higher percentage of their milk quota to the dairy than teams with poor quality teamwork. There is also a tendency that poor teamwork influences cattle health negatively compared to good teamwork. The rich context in a semi- structured interview setting allowed us to get a deeper understanding of the underlying mechanisms. One of the interviewed farmers with low quota filling told us in detail about the collaboration problems:

“Well … you know… since the relations between us has become the way they are [bad]…, it has become a big problem to get the daily work done. We have to rely on occasional labour, and the first thing that suffers then is the production.”

It turned out that bad working climate had reduced the partners’ involvement and willingness to work in the joint operation. This lead to lack of qualified labour, and therefore, they had to rely on occasional labour of varying quality. Quality of teamwork is not registered in cattle-databases. If we had only assessed the production figures and not visited the farm, we would not have fully understood the reasons behind the low quota filling. Neither would we have been in a position to assess what kind of help to offer them. After the interview we were convinced that unless the partners could improve their collaboration, it would be of limited use to offer them extensive services on e.g. preventive health care. The farmers simply needed help to collaborate. This case illustrates the importance of understanding the social context in which the farmers operate. Clearly, without the particular contextual knowledge we could easily make a wrong conclusion in this particular case.

Furthermore, we also conducted in-depth analyses by contrasting different cases. For example, we found the herd fertility on one farm to be extremely good, and the fertility on another farm to be extremely low. Both cases had very low teamwork quality, so teamwork quality cannot explain the difference in fertility between them. However, in-depth analysis of the two cases revealed one important distinction. The manager on the first farm lived at the farm, while the manager on the second farm lived several miles away from the farm. Thus, the first farm manager could easily follow up fertility issues several times a day, while this was much more difficult for the farmer on the first farm. Interestingly, one of the collaborating farmers on the second farm actually lived on the farm. Before entering the joint operation, he had been awarded for having high fertility in his herd. However, due to the high level of conflicts in the joint operation, he just didn’t care about lending a hand there anymore. In general, having a bad relationship reduces the partners’ willingness to engage in interactive problem-solving behaviour (cf. [18]). Indirectly then, the bad social relationships at the second farm may have contributed to the extremely poor fertility at the farm.

## Discussion

The studies discussed above and summarised in Table 1 reflect a growing recognition among veterinarians that traditional, large quantitative studies of cattle databases can only partly explain the variation in health related issues. The traditional approach needs to be supplemented and enriched with knowledge from other fields such as sociology, psychology, management and economics ([13], [9], [11]). We presented three perspectives that can be fruitfully adopted and combined with the biological perspective in agricultural and veterinarian research.

When will these perspectives be most useful? Putting the perspectives into a system, the biological perspective might be seen as the core perspective. The managerial and social perspectives will be especially helpful when we need to explain biological data, while the other three perspectives can explain the economic results of running the farm. Of course this “model” can be reversed, and economic variables may somehow affect biological parameters or management. But we argue that the causal links are more likely to go from the managerial and social perspective variables to the biological variables and further to economic measures.

While recent studies have started to combine the economic or the managerial perspectives with the biological perspective, the social perspective seems to be more or less ignored. This is unfortunate given the vast increase in collaborative farming. The scant research on this perspective indicates that understanding relationship issues is valuable. Social relations are important because collaboration depends on good relations between the parties. If relations are bad, work motivation and willingness to contribute and collaborate will deteriorate, and this will have consequences for several aspects of running a farm. Social relations may also be important in cases where farmers do not run collaborative farms; simply discussing problems and helping the neighbours may have important effects on biological and economic variables.

In conclusion, most veterinary and agricultural research is rooted in the biological perspective. However, in this paper we provided several examples on how information available in cattle-databases can be utilised in a broader sense than what is often the case today. Merging data from the biological, economic, managerial, and social perspectives can enrich our understanding of the farming processes and the veterinary challenges associated with it. Looking forward, we believe more attention should go to the farmer as manager and social actor. Given the continuous changes facing most farmers today, collaboration skills and problem solving abilities will be increasingly important in explaining the success of farming operations (cf. [14]). In short, we need a holistic understanding of the farmer and the farming processes.

Expanding the perspectives to study farm operations also requires new ways of collecting information. Perhaps more important, it calls for broad knowledge in analysing the collected data and merging them with biological data. Thus, we need to engage in cross-disciplinary research-efforts. This situation calls for collaboration between researchers from different professions just as [14, p.7] put it: “The combined approach requires besides skills and interdisciplinary collaboration also openness, reflection and scepticism from the involved scientists, but the benefits may be extended to various contexts both in advisory service and science.” Ideally, we hope the perspectives and illustrations presented in this paper can inspire researchers to adopt multiple foci and to increase the use of multi-method approaches in agricultural and veterinary research.

## Competing interests

The authors declare that they have no competing interests.
